# Multiplex staining depicts the immune infiltrate in colitis-induced colon cancer model

**DOI:** 10.1038/s41598-019-49164-3

**Published:** 2019-09-02

**Authors:** Eliana Pivetta, Alessandra Capuano, Eugenio Scanziani, Lucia Minoli, Eva Andreuzzi, Maurizio Mongiat, Gustavo Baldassarre, Roberto Doliana, Paola Spessotto

**Affiliations:** 1 Molecular Oncology Unit, Centro di Riferimento Oncologico di Aviano (CRO) IRCCS, Aviano, Italy; 2 Department of Veterinary Medicine, University of Milan, Milan, Italy; 3 Mouse and Animal Pathology Lab (MAPLab), Fondazione Filarete, Milan, Italy

**Keywords:** Fluorescence imaging, Acute inflammation

## Abstract

Assessment of the host immune response pattern is of increasing importance as highly prognostic and diagnostic, in immune-related diseases and in some types of cancer. Chronic inflammation is a major hallmark in colon cancer formation, but, despite the extent of local inflammatory infiltrate has been demonstrated to be extremely informative, its evaluation is not routinely assessed due to the complexity and limitations of classical immunohistochemistry (IHC). In the last years, technological advance helped in bypassing technical limits, setting up multiplex IHC (mIHC) based on tyramide signal amplification (TSA) method and designing software suited to aid pathologists in cell scoring analysis. Several studies verified the efficacy of this method, but they were restricted to the analysis of human samples. In the era of translational medicine the use of animal models to depict human pathologies, in a more complete and complex approach, is really crucial. Nevertheless, the optimization and validation of this method to species other than human is still poor. We took advantage of Multispectral Imaging System to identify the immunoprofile of Dextran Sulphate Sodium (DSS)-treated mouse colon. We optimized a protocol to sequentially stain formalin fixed paraffin embedded murine colon samples for CD3, CD8a, CD4, and CD4R5B0 antigens. With this approach we obtained a detailed lymphocyte profile, while preserving the morphological tissue context, generally lost with techniques like gene expression profiling or flow cytometry. This study, comparing the results obtained by mIHC with immunophenotyping performed with cytofluorimetric and standard IHC methods validates the potentiality and the applicability of this innovative approach.

## Introduction

Animal models of inflammation have greatly contributed to our current understanding of several immune-related pathologies, from rheumatoid arthritis to psoriasis as well as cancer, in particular colorectal cancer^1–3^. The density and distribution of local inflammatory infiltrates have been demonstrated to be highly informative in colon cancer^4,5^, but unfortunately, quantitative and specific evaluation is not routinely assessed. On the other hand, general and systemic inflammatory parameters (such as the neutrophil-to-lymphocyte or/and the platelet-to-lymphocyte ratio and the modified Glasgow prognostic score) together with tumour staging represent the most widely used approach in clinical practice^6^. Researchers take mainly advantage of flow cytometry (FACS) to investigate the immunological contest. Cytofluorimetric analysis is the eligible assay to perform immunophenotypic studies on peripheral blood, spleen or bone marrow, tissues from which cells could be easily recovered. Although cells isolated from other tissues, such as lymph nodes or intestine^5^, have been employed for FACS analysis, the protocol is demanding and the results could be affected by processing artefacts^7,8^. On the other hand, immunophenotyping on tissue sections by standard immunohistochemistry (IHC) is generally discouraged, due to technical limits, such as staining protocols and panels, time and material required, low inter-laboratory reproducibility, subjectivity of scoring. IHC approach is poorly feasible when samples are limited and even if several sequential slices are available tissue context reconstruction is sometimes laborious. The application of multiple staining in bright field IHC is challenged by the narrow dynamic range of chromogens, since colour combinations distinguishable by eye from each other is arduous, particularly when looking to co-localized proteins. The use of fluorophores figures out the limitation of IHC in bright field microscopy, since the spectra of several fluorophores could be individually solved with dedicated software analysis programs. However, the limitation of multiple staining in brightfield IHC as well as in immunofluorescence relies on antibody cross-reactivity. The tyramide signal amplification (TSA) detection method, a relative novel strategy, overcomes the need of antibodies directly conjugated or raised in different hosts. In TSA technology the horse radish peroxidase (HRP), conjugated to the secondary antibody, catalyzes a reaction in which multiple tyramide-fluorophore complexes are covalently bound to tyrosine residues at the site of the antigen or in its immediate vicinity, thus resulting in signal amplification. The covalent nature of this binding allows the removal of primary and secondary antibodies, preserving at the same time the fluorescence signal associated with the specific antigen^9^. This method allows performing multiple sequential staining without the concern of cross-reactivity. Multiplex IHC (mIHC) strategies have been demonstrated to be effective by several researchers not only in human^10,11^ but also in animal models^12^. Despite some efforts, immunophenotyping with mIHC approach is still predominantly used only for human samples^13,14^ and its use on murine tissues is still far from being widely performed^15^.

The aim of this study was to optimize a multiple staining protocol for murine formalin-fixed paraffin embedded (FFPE) colon samples to investigate, in the context of colon inflammation, the specific lymphocyte infiltrate profile in an animal model characterized by functional and structural alterations of the lymphatic system. We have previously demonstrated that mice mutant for the extracellular matrix protein EMILIN-1 (E1 TG) displayed functionally defective lymphatic vessels^16,17^. Colon cancer is one of the principal cancer types where a functional link between chronic inflammation, tumour microenvironment and progression has been described^18,19^. Inflammation driving the colitis-associated cancer is also related to striking changes in the lymphatic vasculature^20^. Then EMILIN-1 mutated animal model could be appropriate also to study how lymphatic deregulation affects the inflammatory scenario. The results obtained in the murine model have been compared with FACS analysis and standard IHC to validate mIHC potentiality.

## Results

### Evaluation of DSS-treated mouse colon samples by Flow Cytometry

**Figure 1:**
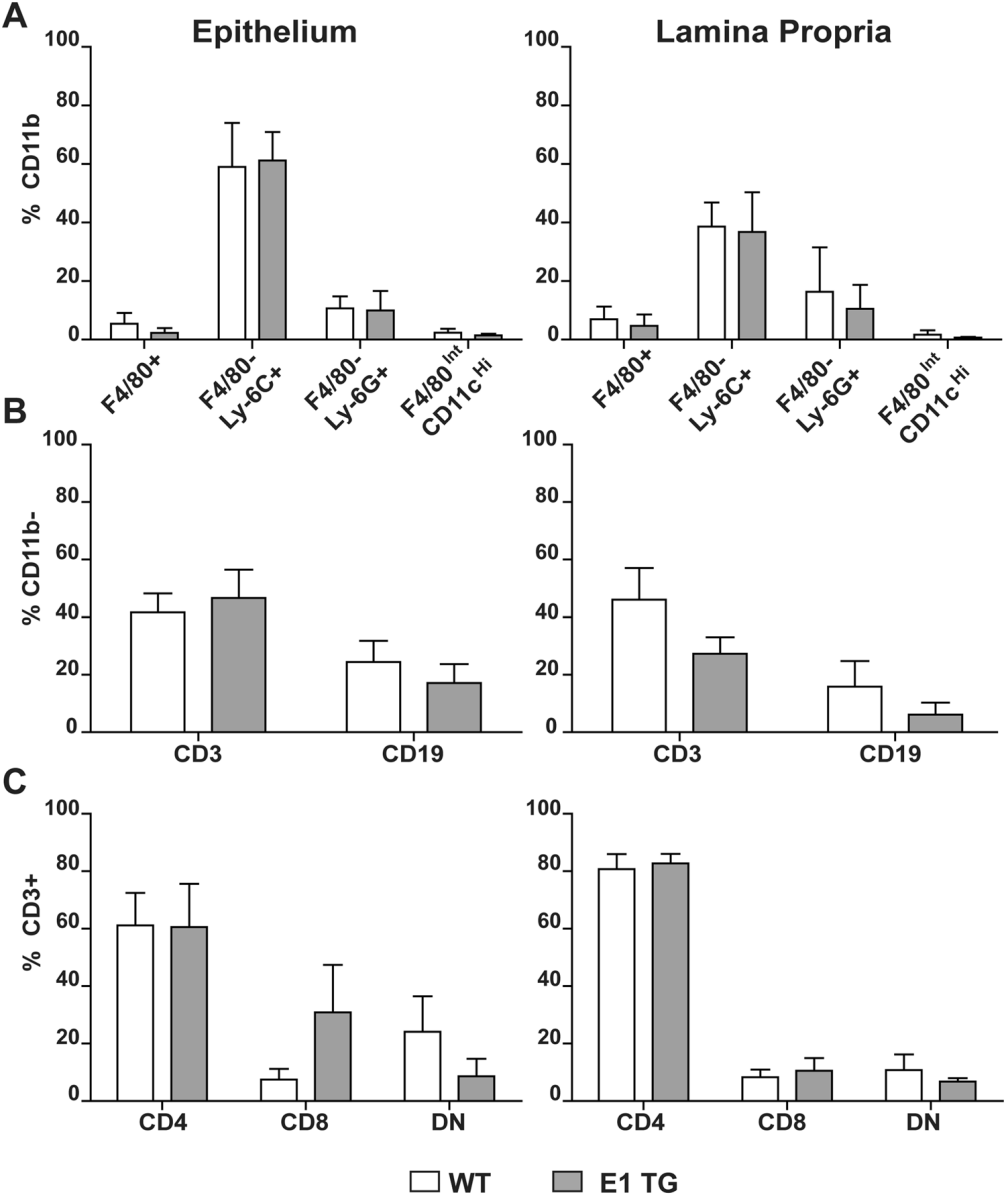
Flow cytometry analysis of immunoinfiltrates in colons. Epithelial (EP) and LP leukocyte population were isolated from colon samples as described in Materials and Methods section. (**A**) Myeloid population was investigated using panel 1 and cells were immunophenotyped as follow: macrophages as CD11b+/F4/80+; moncytes as CD11b+/F4/80−/Ly6C+; granulocytes as CD11b+/F4/80−/Ly6G+, dendritic cells as F4/80^int^/CD11c^Hi^. (**B**) Lymphoid population was characterized using panel 2 and immunophenotyped as follow: T lymphocytes as CD11b−/CD3+/CD19−; B lymphocytes as CD11b−/CD3−/CD19+. (**C**) T lymphocytes were immunophenotyped on the basis of CD4+ or CD8+ gates. Bar graphs show mean values ± s.d. from at least three mice individually tested per group.

Lymphatic alterations are a well established feature of human and experimental colitis. It has also been reported that the dysfunction of lymphatic network affects the inflammatory response. We have recently reported that E1 TG mice display several lymphatic alterations^17^, thus representing a useful model to verify if pre-existing lymphatic dysfunction could determine the progression of DSS-induced inflammation. Chronic inflammation was induced in WT and E1 TG mice by administration of DSS in drinking water (Fig. S1). The inflamed colons were processed as follows: one portion was subjected to collagenase digestion, Epithelial Portion (EP) was isolated from Lamina Propria (LP) fraction and the isolated cells were labelled for FACS analysis; the remaining portion was fixed and paraffin embedded to perform classical and multiplex IHC (Fig. S1). By FACS, immune infiltrates were identified on the basis of hematopoietic CD45 marker, to distinguish blood from stromal and epithelial cells. CD11b+ cells were immunophenotyped on the basis of panel 1 (see Materials and Methods) as: macrophages (F4/80+), monocytes (Ly6C^Hi^/Ly6G^Low-Neg^), granulocytes (Ly6C^Low-Neg^/Ly6G^Hi^), and dendritic cells (F4/80^Int^/CD11c^Hi^). In general, we didn’t observe differences in colon samples in terms of CD45+ cells between WT and E1 TG mice (data not shown). The macrophages were slightly but not statistically decreased in E1 TG versus WT mice, in the EP and LP fractions (Fig. 1A). Vice versa, we didn’t observe differences in E1 TG respect to WT colon samples in terms of granulocytes, monocytes and dendritic cells both in EP as well as in the LP fraction (Fig. 1A). Lymphocytes were identified using a second panel of markers (panel 2, see Materials and Methods) gating for CD11b- and then for CD3 and CD19. We observed a general decrease of lymphocytes (except for CD3+ in EP) in E1 TG versus WT mice both in EP and LP fractions (Fig. 1B). The differences highlighted a trend but they were not statistically significant. When we characterized the T population for T helper (CD4+) and T cytotoxic (CD8+) cells, we observed a significant increase of CD8+ (P = 0.016) and a decrease in double negative cells (CD4−/CD8−) (P = 0.037) in E1 TG *vs* WT mice in the EP, meanwhile only a trend was observed in the LP fraction (Fig. 1C). No difference emerged in both fractions for CD4+ population (Fig. 1C).

### Evaluation of DSS treated mouse colon samples with standard IHC

**Figure 2:**
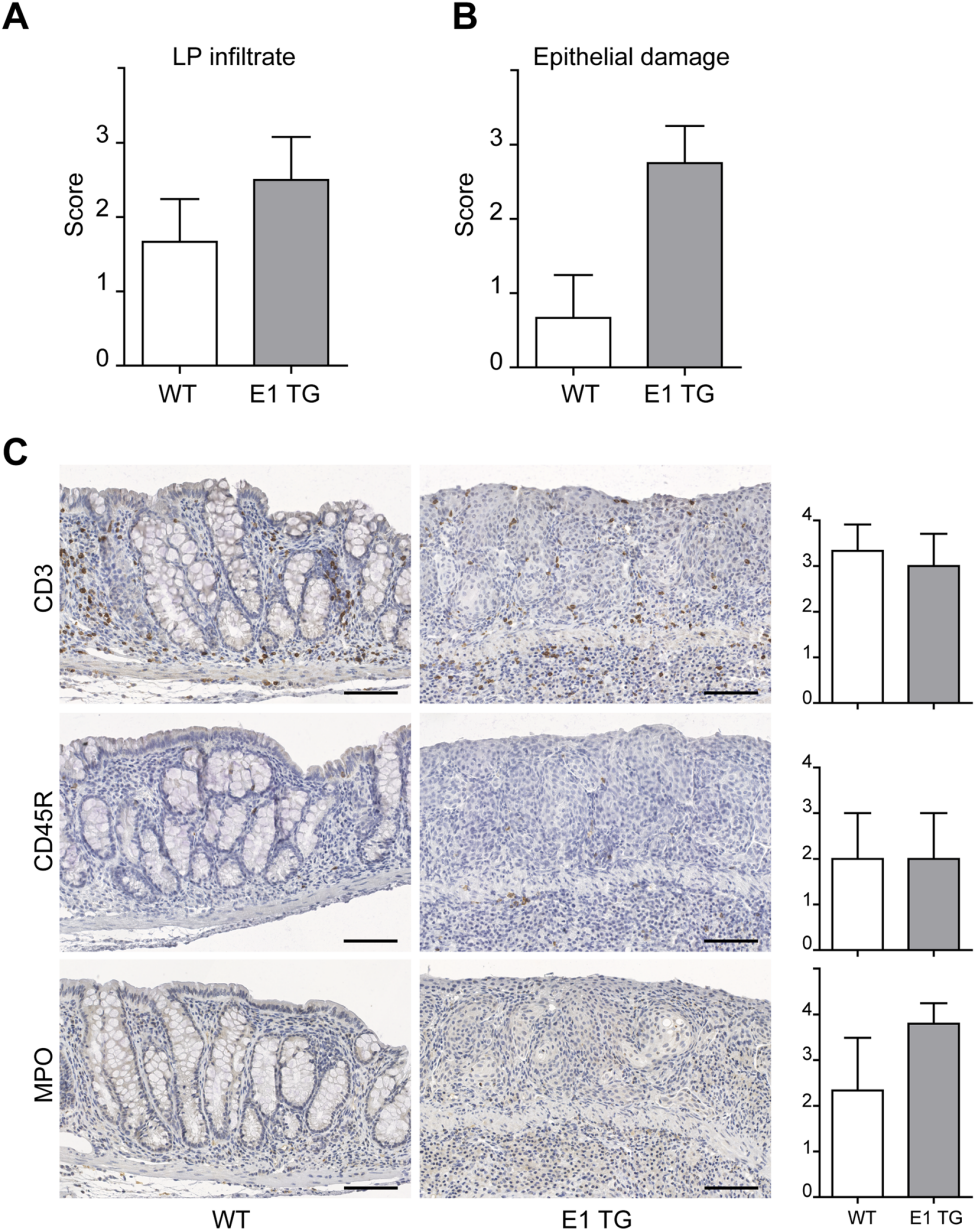
Evaluation of DSS-treated colon with standard IHC. (**A**) The LP infiltrate status was scored as follow: 0 = absence of infiltrating cells; 1 = poor presence of infiltrating cells; 2 = moderate presence of infiltrating cells; 3 = severe presence of infiltrating cells. (**B**) Epithelial damage was determined evaluating the integrity of the epithelial layer of the enteric mucosa. This parameter was scored as 0 = absence, 1 = low, 2 = moderate; 3 = severe. (**C**) Representative images of standard IHC of WT and E1 TG treated colon samples for T (CD3) and B (CD45R/B220) lymphocytes and myeloid cells (MPO). Inflammatory cells were scored counting the number of positive cells within the mucosa (excluding cells of lymphoid follicles): 0 = cell absence; 1 = 1–5 cells; 2 = 6–25 cells; 3 = 26–125 cells; 4 > 125 cells. Bar graphs show mean score values ± s.d. from at least three mice individually tested per group. Scale bar = 100 μm.

Another portion of the same DSS-treated colon sample was scored by standard IHC for LP infiltrates, epithelial damage and inflammatory cells. The resulting analysis revealed a more pronounced inflammatory infiltrates in the LP of E1 TG than in WT mice, even if the difference was not statistically significant (Fig. 2A). The epithelial status assessment revealed a greater damage in E1 TG respect to WT colon tissues (Fig. 2B). To identify the nature of the inflammatory infiltrate, standard IHC was performed to stain for CD3, CD45R/B220, and MPO. The score for the single population infiltrate was achieved counting the number of positive cells within the mucosa, excluding cells of lymphoid follicles. No statistically significant differences were observed scoring for CD3, CD45R or MPO positive cells, but only a positive trend for MPO+ cells in E1 TG mice (Fig. 2C).

### Generation of multiplex protocol

**Figure 3:**
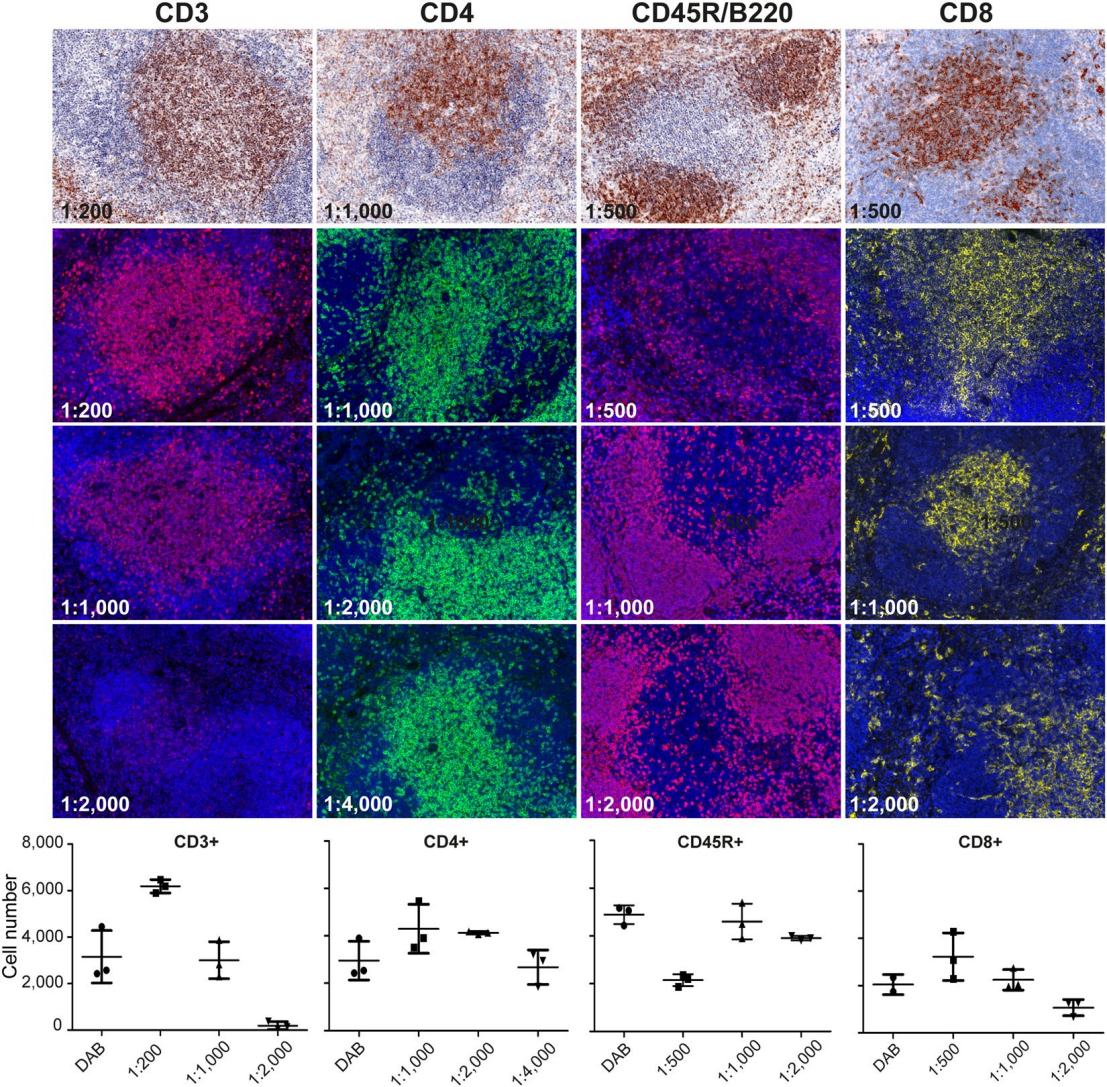
Optimization of monoplex staining. Representative images of spleen sections for the optimization of staining protocols. Images of the dilutions for each antibody starting from the one suggested by the manufacturer as monoplex staining on sequential slides are shown. InForm Cell Analysis was applied on both DAB IHC and TSA IHC images and cells were scored by counting the number of positive cells in three different fields. Original magnification, 200x.

To demonstrate that a detailed immunophenotyping with the preservation of tissue morphology was feasible by performing mIHC, we validated and optimized the staining for each antibody, as a first step. Since the TSA staining technology is more sensitive than conventional DAB or immunofluorescence, it was necessary to dilute primary and secondary antibodies to optimize and balance the signal^21^. Optimal primary antibody concentration was determined on sequential slides of FFPE wild type murine spleen samples, with serial dilutions starting from the one recommended by the manufacturer. To identify the optimal concentration we compared the staining performed with TSA method with the staining visualized by classical IHC (using DAB as a substrate), still considered the gold standard. Staining optimization for CD3, CD4, CD8a and CD45R/B220 antigens is provided in Fig. 3. When we scored cell positivity at the same antibody concentration, the TSA visualization yielded higher positive cell number than DAB method (except CD45R/B220, Fig. 3). We choose the primary antibody dilution at which positive cell number for TSA method correlated with DAB visualization (1:1,000 for CD3; 1:4,000 for CD4, 1:1,000 for CD8a and CD45R/B220).

**Figure 4:**
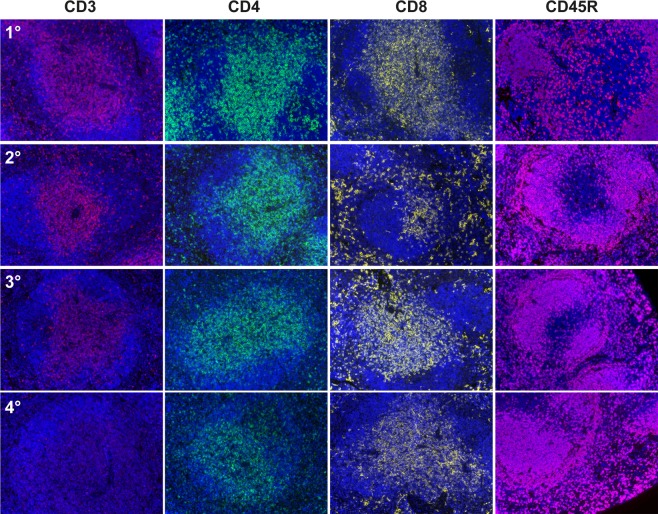
Optimization of primary antibody order for multiplex staining protocol. Representative images of the staining for each marker at different MWT cycles on sequential slides are shown. Incubation with the primary antibody was performed after the first (1°), or the second (2°), or the third (3°) or the fourth (4°) round of MWT. Original magnification, 200x.

Once optimal dilution was achieved, we determined the best antibody order in the staining procedure. To set the positions where the antibodies had to be added, we performed monoplex staining, testing each antibody after different cycles of microwave treatments (MWTs) as depicted in the scheme of Supplementary Fig. S2. The results are reported in Fig. 4. We evaluated the staining efficiency, comparing the fluorescent signal intensity resulting from the different MWT cycles. CD8a antigen could still be detected after three rounds of MWTs (position 3), meanwhile CD3 required the first position because after two cycles the fluorescence intensity and the number of fluorescent cells decreased. CD4 staining was still effective after two MWT cycles, then the signal started to became patchy and the counts started to decrease (position 2, 3 and 4). CD45R/B220 was the strongest antigen and its staining was still efficient after four rounds of MWTs (position 4).

A possible false or unspecific signal due to the unbalanced use of secondary antibodies (especially in mIHC) was excluded by performing IHC omitting primary antibodies and staining with TSA or DBA (Fig. S3). In both cases we showed that the detection method was specific. Even if FFPE samples retained autofluorescence signal, final pure and suitable images were produced (images a–d versus a’–d’ in Fig S3B) by the use of the multispectral image analysis software, able to subtract tissue autoflorescence, as previously confirmed by others^22^.

**Figure 5:**
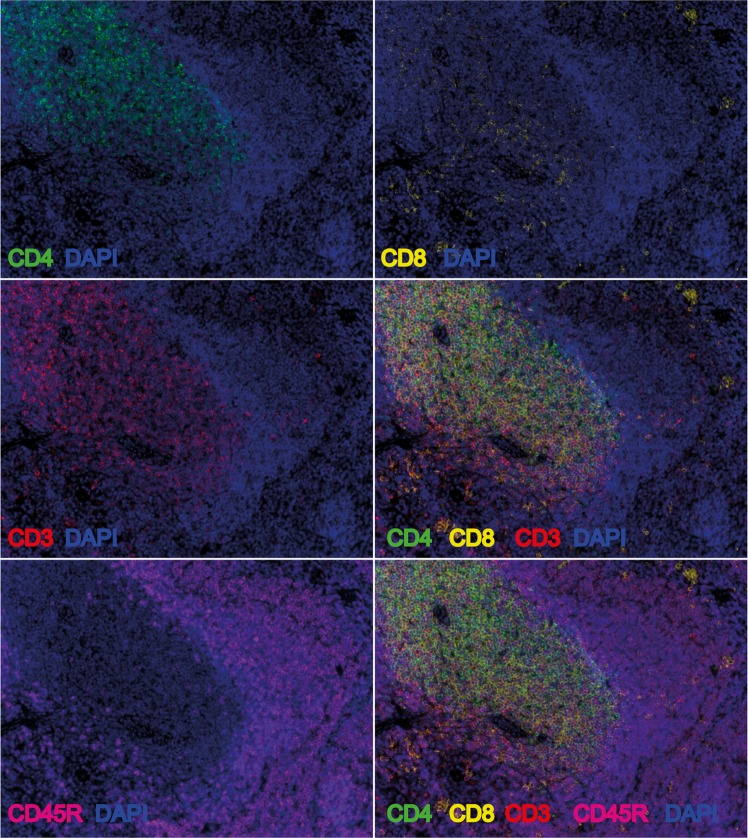
Validation of the multi-staining protocol. The optimized multi-staining protocol was applied on spleen FFPE murine sections. Slides were stained with CD3 (1°, OPAL620, red), CD4 (2°, OPAL520, green), CD8 (3°, OPAL540, yellow) and CD45R/B220 (4°, OPAL690, magenta) antibodies. Representative images of each single channels, of CD3-CD4-CD8 merge and CD3-CD4-CD8-CD45R merge are shown. Original magnification, 200x.

**Figure 6:**
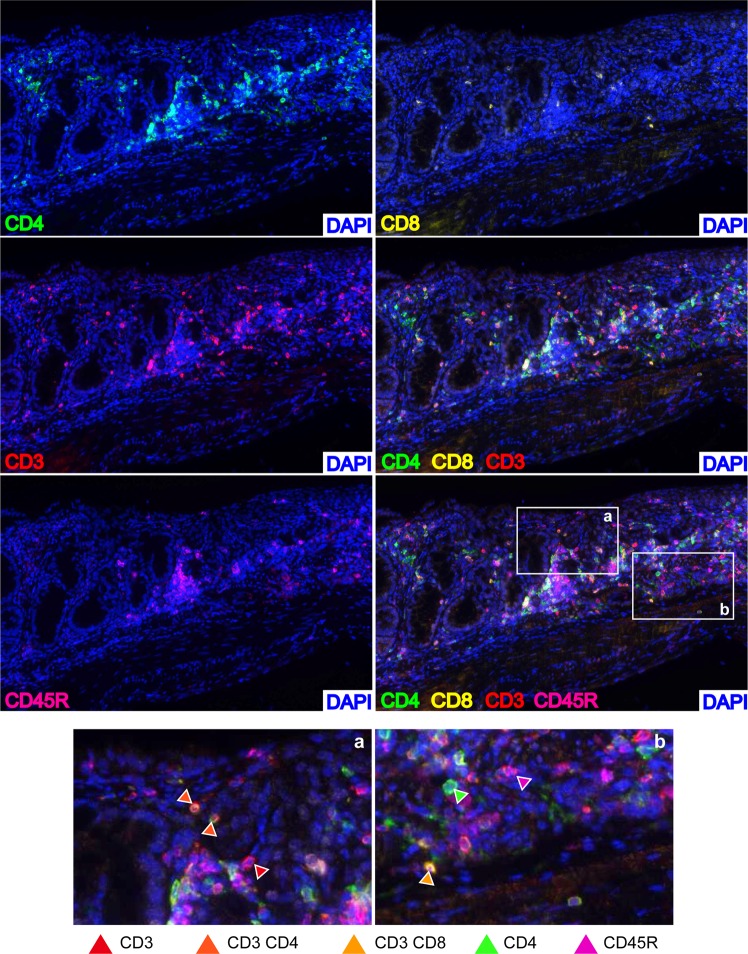
Application of the multi-staining protocol. The optimized multi-staining protocol was applied on colon FFPE murine sections. Slides were stained with CD3, CD4, CD8a and CD45R/B220 antibodies. Representative images of each single channel, of CD3-CD4-CD8a merge and CD3-CD4-CD8a-CD45R/B220 merge are shown. Inset on the left, single CD3+ (red), double CD3+CD4+ (dark orange); double CD3+CD8a+ (orange), single CD4+ (green), single CD45R/B220+ (magenta). Original magnification, 200x.

We then combined the monoplex into a multiplex protocol and we verified the feasibility first on control tissue (spleen, Fig. 5) and then on colon DSS-treated samples (Fig. 6). Our mIHC was clearly able to discriminate T (CD3) and B (CD45R/B220) lymphocytes and to immunophenotype CD4 and CD8a positive cells (Fig. 6, insets).

### Evaluation of DSS treated mouse colon samples with multiplex staining

**Figure 7:**
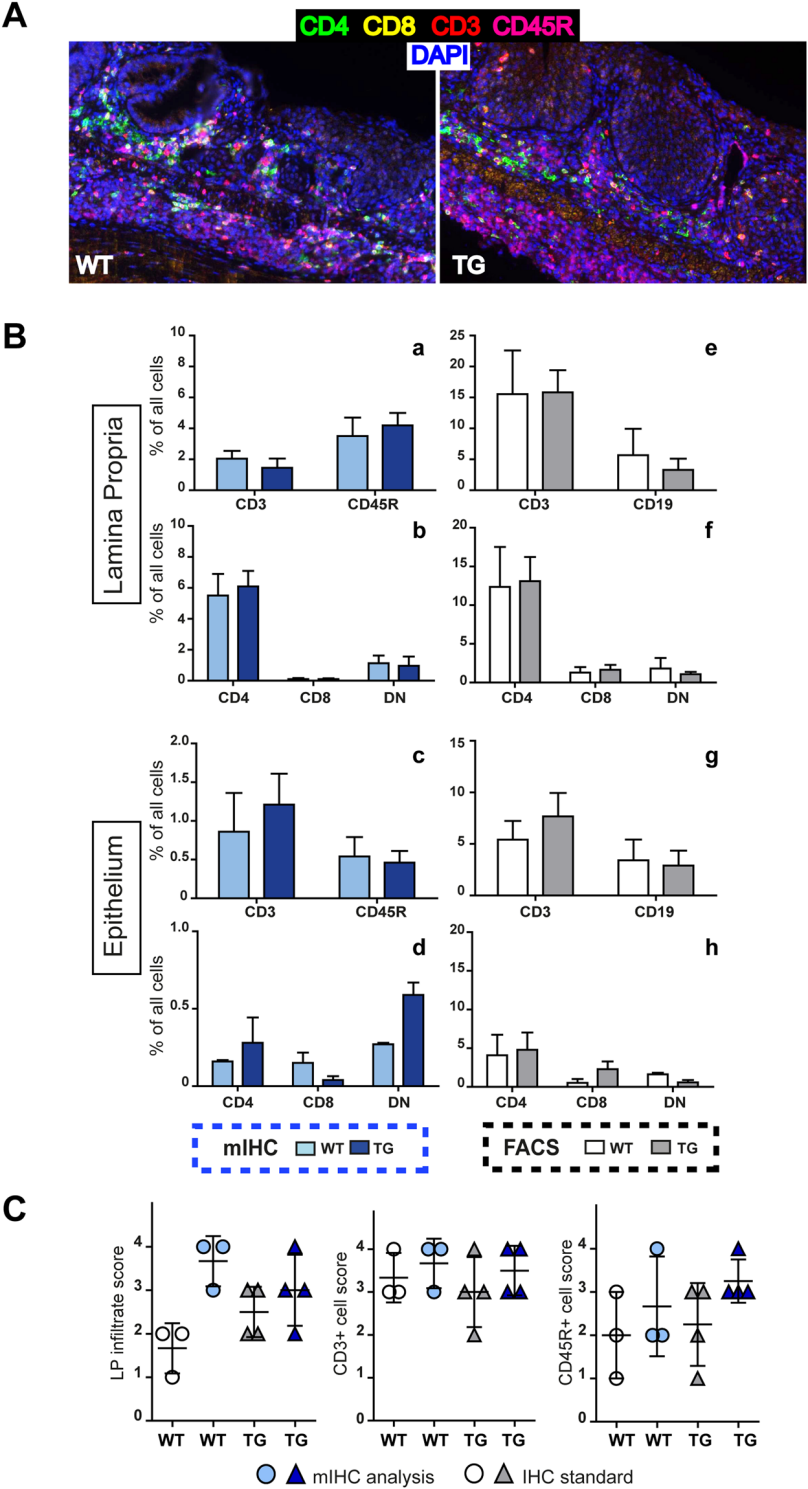
Comparison of multi-staining protocol with FACS and standard IHC techniques. (**A**) Representative images of WT and E1 TG DSS-treated colons multiple stained for CD3 (red), CD4 (green), CD8a (yellow) and CD45R (magenta). Original magnification, 200x. (**B**) Leukocyte analysis of the LP (a and b) and EP (c and d) fractions performed with mIHC, calculating the percentage of each population on the number of total cells (on the left); leukocyte analysis of the LP (e and f) and EP (g and h) fractions performed with FACS, recalculating the percentage of each population on the total events (on the right). (**C**) Comparison between mIHC with standard IHC results. We converted the number of positive cells coming from inForm software analysis into the same score applied in the histology evaluation as reported in Fig. 2. Bar graphs show mean values ± s.d. from at least three mice individually tested per group.

To characterize the inflammatory infiltrate in WT and E1 TG mice, we applied the optimized protocol of multiple staining on FFPE murine colon samples. In Fig. 7A, mIHC clearly and specifically identified lymphocyte infiltrate in both WT and TG samples. Analysis was performed on acquired multispectral images using dedicated software inForm, that allowed to distinguish different tissue categories, such as stromal and epithelial tissue component, and to identify and quantify cell scoring for marked populations. We focused on lymphocyte analysis, quantifying T (CD3+) and B (CD45R/B220+) percentage and number, phenotyping T cell subpopulations (CD4+ and CD8a+) and evaluating their distribution in tissue categories (Fig. 7B, a–d). To compare the results obtained by FACS and mIHC, we converted the cytofluorimetric cell percentage (Fig. 1) normalizing the number of positive cells to total events (considered as total extracted cells) (Fig. 7B, e–h). The difference in the absolute value of percentage was explained considering that the FACS analyses were performed on total tissue, whereas the mIHC analysis selectively excluded the leukocytes of lymphoid follicles. Generally, we observed a concordance of results between the two approaches, even if the mIHC provided a lower percentage number; the only difference between the two analyses regarded the CD8 and double negative percentages in the EP fraction (Fig. 7B, d and h). Thus, our mIHC could identify and quantify lymphocyte subpopulations providing results quantitatively comparable to flow cytometry.

The dedicated software for multispectral image analysis counts every positive cell and provides the relative percentage for a specific biomarker in each tissue category, and the total (positive and negative) cell number per field. We used the resulting data to calculate the number of total positive cells. In order to compare the mIHC to classical IHC, we converted the number of positive cells into the same score applied in the histology evaluation of Fig. 2. In general, for every sample our score was higher than that assigned by the pathologist (Fig. 7C). Notwithstanding, the results were broadly consistent between the two approaches, showing the same grade for each population.

By the use of this protocol, cells were not only immunophenotyped on the basis of negative/positive staining, but their contextual localization was preserved. Thus, we were able to qualitatively and quantitatively characterize the immune infiltrate in an intact and native microenvironment.

## Discussion

The complexity and limitations of classical IHC (technical limits, different staining protocols and panels, low reproducibility, subjectivity of scoring) have for long times discouraged the immunophenotyping of tumour infiltrates. Nevertheless, in the last years, technological advances have created the basis to bypass these limitations. “mIHC” identifies all the technologies that permit to detect multiple biological markers on a single tissue section. mIHC based on TSA method circumvented the technical restrictions due to classical IHC and supportive and dedicated software packages have been created to aid pathologists in cell scoring analysis, overcoming the problematic of manual scoring methods and increasing objectivity, accuracy and reproducibility.

The use of animal models to study the molecular and cellular mechanisms underlying several pathologies has a high impact in translation research; for this reason it is crucial to optimize methods and techniques entailing mouse tools. Despite mIHC is an effective and supportive method, its application is still mainly restricted to human samples^23–25^. Several concerns have to be taken in consideration. Antibodies against CD mouse antigens are increasing in the last time, but their use on FFPE samples is still not well validated. To date, reliable and consistent identification of specific CD markers, such as CD4 or CD8, by IHC in FFPE mouse tissues has been difficult and mainly performed on frozen sections, where the tissue architecture is not always preserved, thus discouraging morphological and immune profile studies. Indeed, few studies dealt with the application of mIHC approach to analyse multiple biomarkers in murine cryostat or FFPE tissues^9,12,15,26–28^. Feng *et al*.^15^ described a detailed and well planned protocol to performed mIHC on FFPE murine spleen, using zinc-fixative based protocol that unfortunately is not routinely performed. Recently, Sorrelle *et al*.^27^ validated several antibodies for the establishment of an improved multi-staining protocol of FFPE murine spleen and liver samples; nevertheless, they didn’t deeply take advantage of the multispectral system potentiality, because their approach was limited by the number of available filter channels, in some cases staining two markers with the same fluorophore. Our study is the first that validated and optimized a multi-staining protocol for FFPE mouse colon tissues to identify lymphocyte population. Moreover, in a relative short staining schedule time every antigen was unequivocally identified by a single fluorophore without any extensive management of the slides. The procedure required titration of each antibody and once optimal dilution was achieved, we determined the sequential order of antibodies. This is a critical step, because multiple rounds of MWT could compromise target specific antigens, hampering the detection, or vice versa could expose more some epitopes, providing higher signal^21^. Moreover, when two epitopes are spatially very close (nanometers), because they contribute to the formation of a receptor complex, such as the case of CD3 with CD8 or CD4, interference is possible, and should be assessed.

The principal aim of our study was to verify if a multi-staining protocol could be a useful and suitable approach to investigate the infiltrate profile in a contest of a colitis-induced colon cancer using an animal model where lymphatic alterations could regulate the inflammatory composition. It has been demonstrated that not only the density (cell number) but also the histological distribution (cell localization) could impact on colon cancer clinical stage and outcome^4,29,30^. Even if the positive prognostic value of cytotoxic CD8 T cells has been widely accepted in colon cancer, the incidence and distribution of other lymphocytic subpopulations could unbalance the immune surveillance. We demonstrated that it was possible with our multi-staining protocol to clearly identify the main lymphocytic populations (B and T cells, and this last declined in CD4 and CD8a), using a multiplex staining protocol (Figs 5 and 6); we obtained results which were comparable to FACS analysis (Fig. 7A), that is considered the gold standard for immunophenotyping. We observed slight differences in cell distribution and discordance in B cell number and percentage. One possible explanation is dependent of the fact that the mIHC analysis selectively excluded the leukocytes of lymphoid follicles, rich in B cells, whereas the FACS analyses were performed on total tissue. Taken together, these data indicated that the lymphatic vessel alterations of our E1 TG mice did not affect the specific lymphocytic infiltrate investigated. We cannot exclude that other leukocyte populations could affect the progression of colon cancer in a compromised lymphatic context and future investigations will extend the field of analysis to myeloid populations, where some differences were highlighted by FACS, optimizing specific mIHC protocol for this purpose.

In conclusion, the spatial relation of inflammatory cells in pathologic tissues is a very relevant parameter to define the active role of immune infiltrate. Thus, even if FACS analysis provides a detailed immunophenotyping, the tissue assessment status is completely lost; vice versa the standard IHC, that is the benchmark technique, well describes architecture alterations but the immunoscore flattened little differences. Moreover, classical IHC requires several slides for each sample to perform an accurate analysis of the inflammatory infiltrate. It is evident that the mIHC technique solves this limitation combining in a single section the detection of different leukocyte populations. In addition, the possibility to objectively count every single labelled cell permits to overcome the score approach and to report in a quantitative manner each marked population, highlighting fine differences, and defining the histological distribution of the infiltrate in each tissue category.

## Materials and Methods

### Mice

All animal procedures and their care were performed according to the institutional guidelines, approved by the CRO-IRCCS Organismo Preposto al Benessere degli Animali (OPBA, i.e. Committee for the Animal Wellbeing) in compliance with the provisions of EU directive (2010/63/UE) and national laws and authorizations by the Italian Ministry of Health to Dr. Spessotto (n. 248/2015) and to Dr. Mongiat (n. 148/2016). Female FVB mice were purchased from Charles River Laboratories. E1 TG mice (FVB background) were generated as previously described^17^. These mice express EMILIN-1 with a mutation (E933A) that abolish the interaction with its integrin receptor. 6–8 weeks old female were randomly divided in control and treatment groups (3 to 4 animals per group).

### Induction of chronic colitis by DSS

For induction of chronic colitis, mice were administered 3% (weight/volume) DSS (molecular weight 36–50 kilodaltons; MP Biomedicals, France) in drinking water. Fresh DSS solution was prepared daily. The schedule administration was described in Supplementary Fig. S1. The body weight, survival rate and disease activity index were^31^. Mice were anesthetized with an intraperitoneal injection of ketamine (Imalgene, Merial, Italia) (100 mg/kg) and xylazine (Rompun, Bayer, Germany) (10 mg/kg) and monitored by colon endoscopy after every DSS treatment cycle to directly visualize DSS-induced colonic mucosal damage *in vivo* (Coloview system, Karl Storz Veterinary Endoscopy, Germany). At the end of experiments mice were euthanized, colons were removed, washed with saline solution, and measured. Colon samples were cut and equally divided for flow cytometry, standard and mIHC analysis.

### Cell isolation and flow cytometry analysis

Flow cytometry was performed on single cell suspension of colon cells isolated as previously described^32^. Briefly, after mice were euthanized, the colon was harvested, arranged in a “jelly roll” and then the organ was inflated with cold PBS to solubilise tissue contents. Then the organ was opened longitudinally and cut into pieces, placed in a 50 ml conical tube containing RPMI-1640 Medium (Sigma, Italy) and stored on ice. For isolation of epithelial cells, tissue pieces were incubated with EDTA solution (5 mM EDTA/2% FBS in HBSS) and vigorously shacked. Washes were filtered through a 70 μm cell strainer to retain epithelial cells (BD Biosciences, Italy). Following epithelial cell removal, tissues were transferred on Petri dish, minced into fine pieces and incubated with Liberase (Roche, Switzerland) solution (0.2 Wünsch U/ml Liberase, 200 U/ml Dnase) to perform digestion at 37 °C for 30 min, mixing every 10 min. RPMI was added to stop the collagenase activity. Samples were passed through an 18 gauge needle and filtered through a 70 μm cell strainer. Cells were centrifuged at 300 × *g* for 5 min, washed twice in 2% FBS in HBSS, resuspended in RPMI and stored on ice. Cells were incubated with the following antibody panels: **panel 1)** CD45, CD11b, CD11c, F4/80, MHC II, Ly-6C, and Ly-6G; **panel 2)** CD45, CD11b, CD3, CD4, CD8, CD19, and MHC II. Flow cytometry was performed with the FACS LSRFortessa (BD Biosciences) and data were analyzed using DIVA software (BD Biosciences). The complete list of antibodies is present in Supplementary Materials and Methods (Table S1).

### Immunohistochemistry

Colon and spleen samples were fixed in 10% formalin-buffered solution for 24 h. Then they were paraffin embedded and cut to obtain 5 μm sections. For classical IHC, dewaxing, rehydration and antigen retrieval were performed in a single step method: sections were immersed for 40 min at 94 °C in a pH 9 buffer solution (Dewax and HIER Buffer H, Thermo Fisher Scientific). Endogenous peroxidase activity was blocked by incubating samples in 3% H_2_O_2_ for 15 min at room temperature (RT). Slides were rinsed, blocked with 10% normal serum in PBS for 30 min and then incubated for 1 h at RT with primary antibody (see Table S2). An appropriate biotinylated secondary antibody (Vector Laboratories, CA, USA) was added for 30 min at RT and then sections were labelled with avidin-biotin-peroxidase procedure with a commercial immunoperoxidase kit (Vectastain Standard Elite, Vector Laboratories). The immunoreaction was visualized with 3,3′-diaminobenzidine substrate (DAB, Vector Laboratories) for 5 min and slides were counterstained with Mayer’s haematoxylin. For mIHC, FFPE spleen and colon samples sections were deparaffinised and rehydrated. MWT was performed in appropriate antigen retrieval buffer (AR6 or AR9 buffer, accordingly to primary antibody requirements, PerkinElmer, MA, USA). Slides were blocked for 1 h at RT with 5% normal serum in TBS followed by primary antibody incubation at RT for 1 h. Slides were washed three times for 5 min in TBST and incubated with appropriate secondary HRP-conjugated antibody for 30 or 60 min at RT. TSA visualization was performed with OPAL 4-Color Manual IHC Kit (NEL810001KT, Perkin Elmer) containing the following fluorophores: Opal 520, Opal 540, Opal 620, and Opal 690. Slides were incubated for 10 min at RT in the dark, followed by washes with TBST. Multiplex were performed by repeating staining cycles in series, with MWT between each step to remove the antibody complex. For the last step, slides were counterstained with Spectral DAPI reagent for 30 min in the dark at RT (NEL810001KT, PerkinElmer), washed and mounted with Mowiol containing 2.5% DABCO. Multiplex staining protocol was optimized starting from monoplex staining and then performing duplex, triplex and 4plex. For a complete list of antibodies used in this study see Table S2 in Supplementary Materials and Methods.

### Histopathology evaluation

Histopathology evaluation was made in a blind fashion (i.e. without knowledge of the treatment group) on sequential slides of the same samples by an independent laboratory by means of eosin and hematoxylin staining. The following findings have been detected and scored: epithelial damage, LP inflammatory infiltrate (for detailed description see Supplementary Material and Methods).

### Tissue imaging and analysis

Slides were acquired using the MANTRA System (Mantra 1.0.2, PerkinElmer). Immunohistochemical images were acquired in brightfield mode and analysed using inForm Advanced Image Analysis software (inForm 2.4.1, PerkinElmer) upon acquisition and building of absorbance spectra library for DAB and hematoxylin. Multispectral fluorescent images were unmixed using spectral libraries built from images of monoplex stained slides for each OPAL fluorochrome and DAPI, using the inForm software (inForm 2.4.1, PerkinElmer). Multistained images were analyzed with inForm software by the mean of tissue segmentation, cell segmentation, and positive score. At least three fields at 20x magnification were acquired for each sample.

### Statistical analysis

Statistical analysis was performed with GraphPad Prism 5. Statistical analysis comparing two groups were performed using unpaired Student’s t-test, whereas one-way or two-way ANOVA test was applied to properly compare more groups and automatically corrected by Bonferroni’s multiple comparison test. P-values < 0.05 were considered to be statistically significant. All P-values correspond to two-sided significance tests.

## Supplementary information

Supplemental information
