# Supplementary material for: Multiplex staining depicts the immune infiltrate in colitis-induced colon cancer model
Source: Sci Rep. 2019 Sep 2;9:12645. doi: 10.1038/s41598-019-49164-3 (PMC6718623; doi:10.1038/s41598-019-49164-3)
Supplement: Supplementary file 1 — Supplemental information [file 41598_2019_49164_MOESM1_ESM.pdf]

Supplemental material

"Multiplex staining depicts the immune infiltrate in colitis-induced colon cancer model"

Eliana Pivetta, Alessandra Capuano, Eugenio Scanziani, Lucia Minoli, Eva Andreuzzi, Maurizio Mongiat, Gustavo Baldassarre, Roberto Doliana, Paola Spessotto

Supplementary Material and Methods

Flow Cytometry Analysis

For Flow Cytometry analysis the antibodies used are reported in the following table.

Table S1. Primary antibodies used in flow cytometry analysis.

**Primary Antibodies**	Fluorophore	RRID	Brand	Host
CD11b	PerCP-Cy5.5	AB_953560	eBiosciences	rat
CD4	Alexa Fluor 700	AB_494001	Ebiosciences	rat
Ly-6G	FITC	AB_465313	Ebiosciences	rat
CD45	Pe-Cy5	AB_468751	Ebiosciences	rat
CD8	PE	AB_657769	Ebiosciences	rat
CD19	FITC	AB_657666	Ebiosciences	rat
F4/80	BV421	AB_2734779	BD Biosciences	rat
Ly-6C	Alexa Fluor 700	NA	BD Biosciences	rat
CD11c	PE	AB_2033996	BD Biosciences	hamster
MHC II	BV650	NA	BD Biosciences	rat
CD3	BV510	NA	BD Biosciences	hamster

NA = not applicable (in the data base the corresponding antibodies were not found)

Immunohistochemistry

For immunohistochemistry analysis the antibodies used are reported in the following table.

Table S2. Antibodies used in IHC analysis

**Primary antibodies**	Company	RRID	Dilutions	Host	Detection method
CD3e	Abcam	AB_305055	1:1,000	rabbit	TSA
CD3e	Santa Cruz	AB_631128	1:2,000	goat	DAB
CD4	Abcam		1:1,000<br>1:4,000	rabbit	DAB<br>TSA
CD8a	Synaptic System	AB_2800529	1:500<br>1:1,000	rabbit	DAB<br>TSA
CD45R/B220	BD Pharmigen	AB_2737894	1:500(DAB)<br>1:1,000(TSA)	rat	DAB<br>TSA
MPO	Dako	AB_2335676	1:2,000	rabbit	DAB
**Secondary antibodies**					
Anti-rabbit	Vector		1:3	goat	TSA
Anti-rat	Thermo Fisher Sc.<br>Vector		1:500<br>1:200	donkey<br>rabbit	DAB<br>TSA
Anti-goat	Vector		1:200	rabbit	DAB

Histopathology evaluation

Histopathology evaluation was made in a blind fashion, i.e. without knowledge of the treatment group. The following findings have been detected and scored.

<u>Epithelial damage</u>: loss of the epithelial layer of the enteric mucosa. This finding was scored as follow: 0 = absence of epithelial damage; 1 = low epithelial damage; 2= moderate epithelial damage; 3 = severe epithelial damage. (In some cases squamous metaplasia of the epithelial layer was observed.

This finding was scored as epithelial damage with the indication of metaplasia.)

Lamina propria (LP) infiltrate: presence of infiltrating inflammatory cells within lamina propria. This finding was scored as follow: 0 = absence of infiltrating cells; 1= poor presence of infiltrating cells; 2 = moderate presence of infiltrating cells; 3 = severe presence of infiltrating cells

Immunohistochemistry evaluation

Immunohistochemical evaluation was made in a blind fashion, i.e. without knowledge of the treatment group. Immunostaining for inflammatory cells (CD45R/B220, CD3 epsilon, MPO) was scored counting the number of positive cells within the mucosa (excluded cells of lymphoid follicles) as follows: 0 = 0; 1 = 1 to 5 cells; 2 = 6 to 25 cells; 3 = 25 to 125 cells; 4 = > 125 cells.

Supplementary Figures

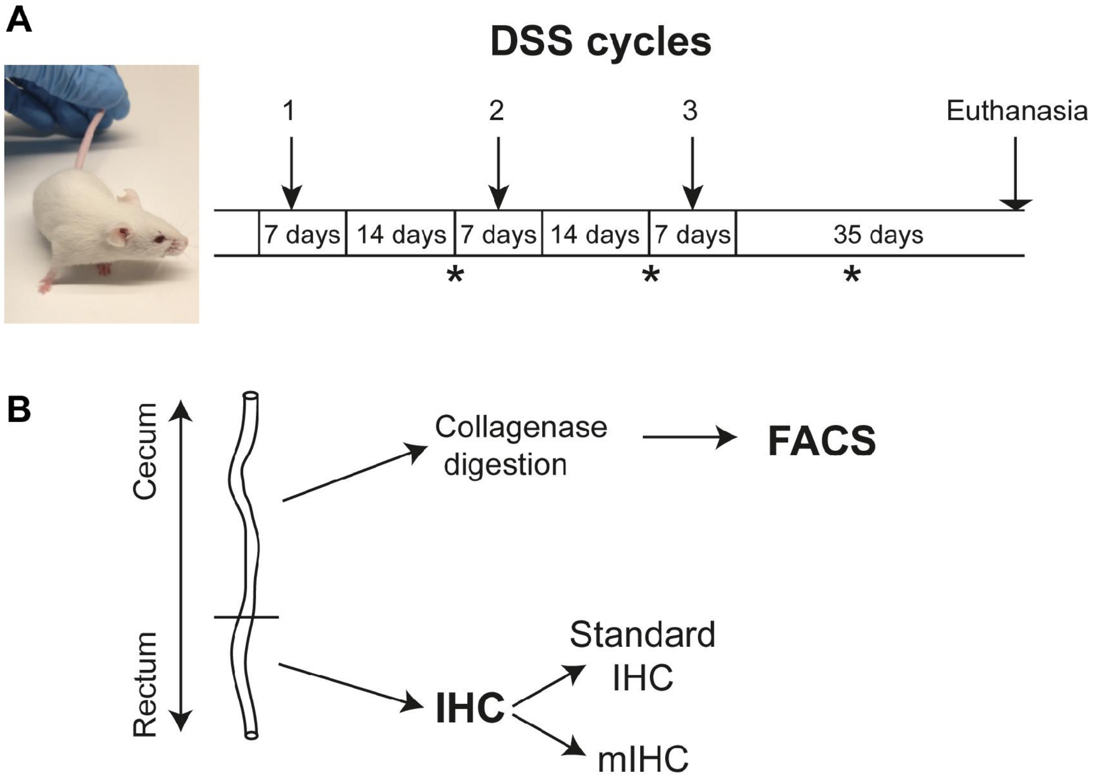

Supplementary Figure S1. Experimental design. A) Schematic representation of DSS treatment to induce chronic colitis. Mice were treated with 3% DSS in drinking water for seven days, then an interval of fourteen days took place. The treatment was repeated for three times. Every 21 days from the beginning, colon endoscopic evaluation was performed (*). B) After euthanasia colons were washed and cut in two portion, one subjected to collagenase digestion for cell isolation and flow cytometry analysis, and one fixed and paraffin embedded for immunohistochemistry analysis.

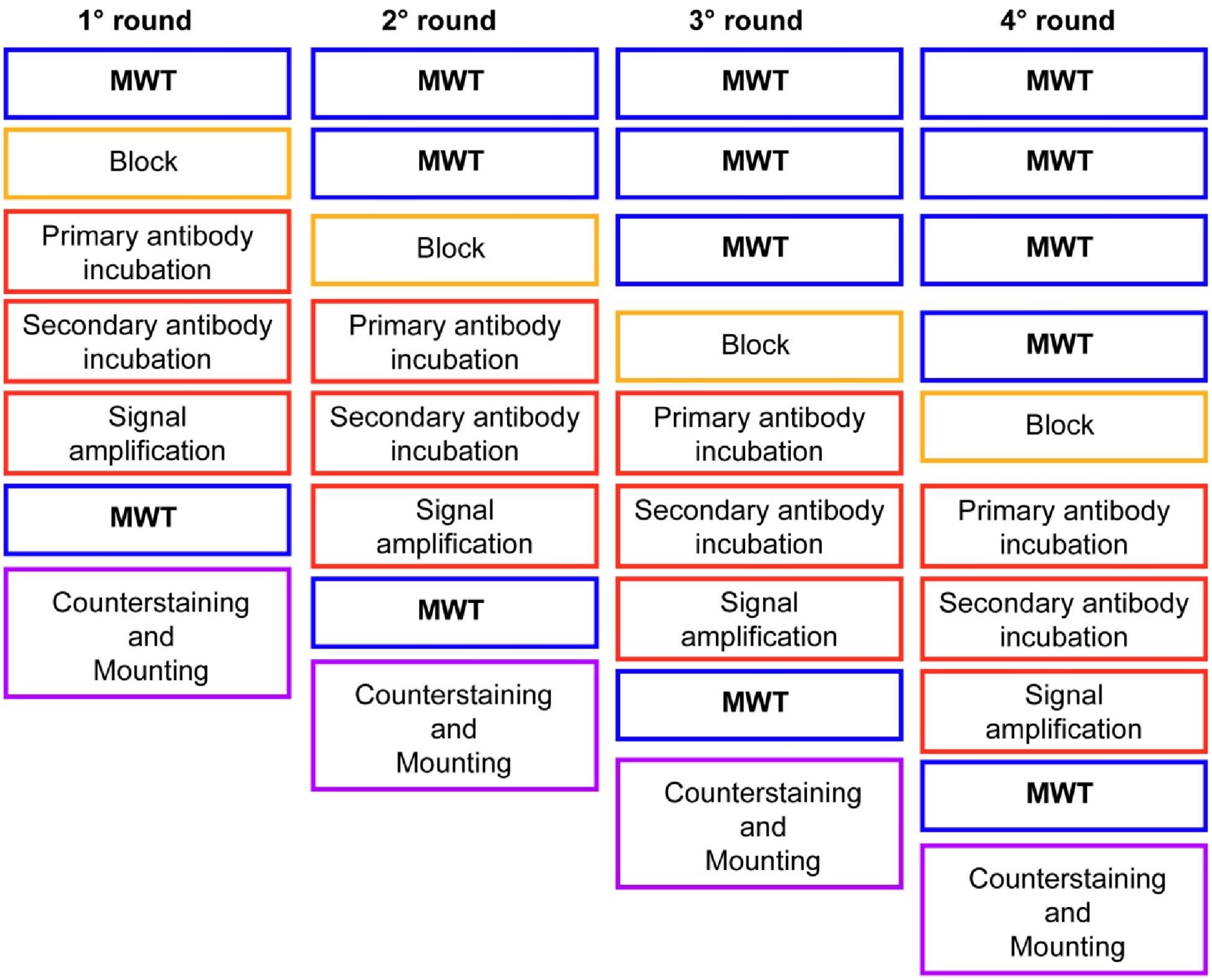

Supplementary Figure S2. Schematic representation to identify antibody sequence incubation. Graphical description of the protocol to test the effect of microwave treatment (MWT) on antigen stability, applied to each antibody used. In classical immunohistochemistry, primary antibody was applied after one round of MWT (1° column). We tested the efficiency of antibody recognition after two, three of four rounds of MWT (respectively 2°, 3° and 4° column).

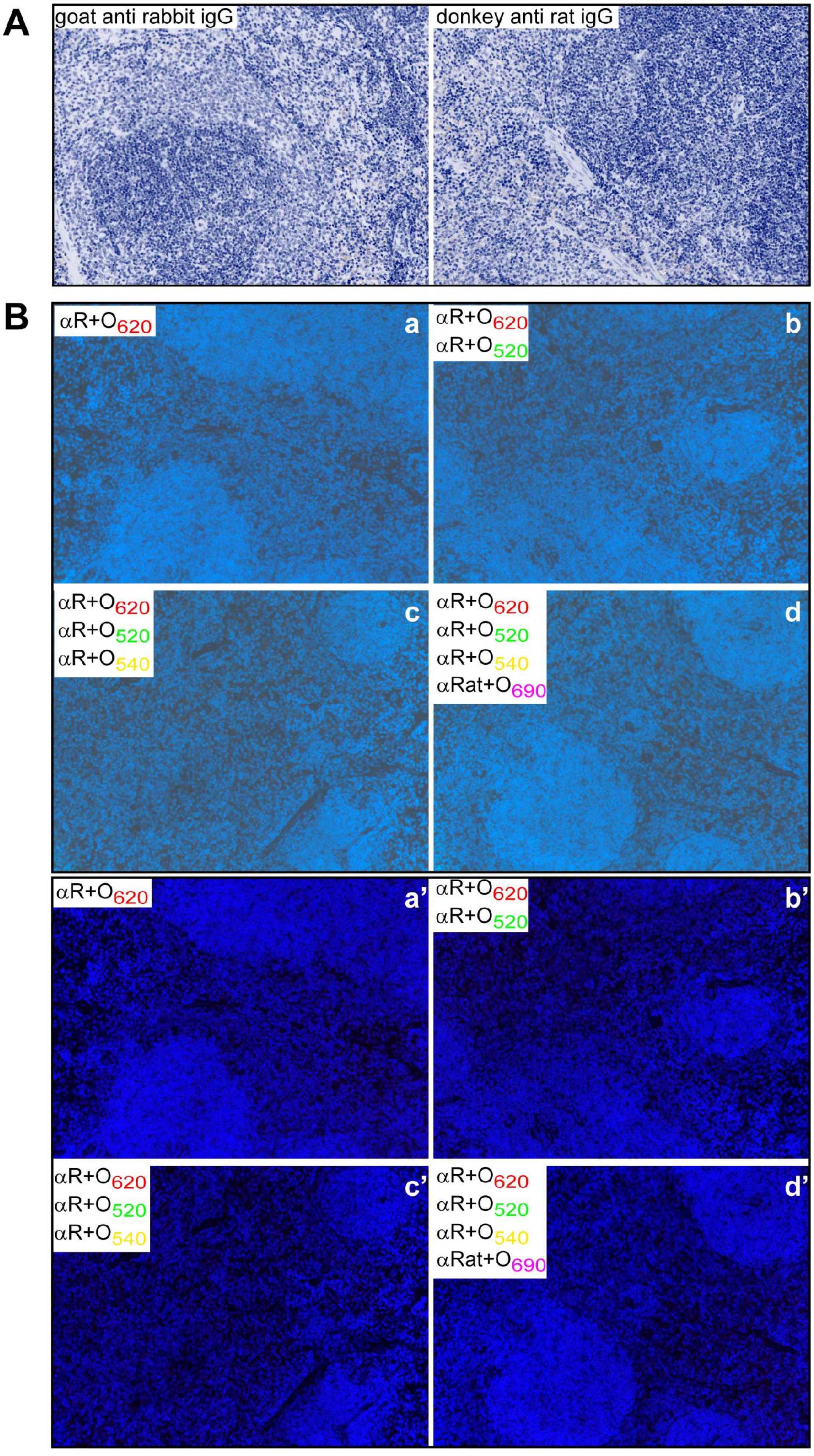
A
goat anti rabbit igG
donkey anti rat igG
B
a
αR+O620
b
αR+O620
αR+O520
c
αR+O620
αR+O520
αR+O540
d
αR+O620
αR+O520
αR+O540
αRat+O690
a’
αR+O620
b’
αR+O620
αR+O520
c’
αR+O620
αR+O520
αR+O540
d’
αR+O620
αR+O520
αR+O540
αRat+O690

Supplementary Figure S3. Negative controls. A) Representative images of FFPE spleen mouse sections processed omitting primary antibodies. The sections were stained using anti rabbit (left) or anti rat (right) HRP-conjugated secondary antibodies followed by DAB detection. B) Representative images of FFPE spleen mouse sections without incubation with primary antibodies and processed using HRP-conjugated secondary antibodies as follows: in a and a', anti rabbit and OPAL620 (mimicked single staining); in b and b', anti rabbit and OPAL620, followed by staining with anti rabbit and OPAL520 (mimicked double staining); in c and c', anti rabbit and OPAL620, followed by staining with anti rabbit and OPAL520 and then by anti rabbit and OPAL540 (mimicked triple staining); in d and d', anti rabbit and OPAL620, followed by staining with anti rabbit and OPAL520, then by anti rabbit and OPAL540 and finally by anti rat and OPAL690 (mimicked quadruple staining). Images a, b, c, and d are obtained from original acquisition; a', b', c', and d' are derived from autofluorescence subtraction process. 200x, original magnification.
